# Prognostic impact of peak oxygen uptake and heart rate reserve in patients after off‐pump coronary artery bypass grafting

**DOI:** 10.1002/clc.23579

**Published:** 2021-02-25

**Authors:** Abidan Abulimiti, Miho Nishitani‐Yokoyama, Kazunori Shimada, Mitsuhiro Kunimoto, Tomomi Matsubara, Kei Fujiwara, Tatsuro Aikawa, Shohei Ouchi, Yurina Sugita, Kosuke Fukao, Tomoyasu Kadoguchi, Tetsuro Miyazaki, Akie Shimada, Taira Yamamoto, Tetsuya Takahashi, Toshiyuki Fujiwara, Tohru Asai, Atsushi Amano, Hiroyuki Daida, Tohru Minamino

**Affiliations:** ^1^ Department of Cardiovascular Biology and Medicine Juntendo University Graduate School of Medicine Tokyo Japan; ^2^ Sportology Center Juntendo University Graduate School of Medicine Tokyo Japan; ^3^ Cardiovascular Rehabilitation and Fitness Juntendo University Hospital Tokyo Japan; ^4^ Department of Cardiovascular Surgery Juntendo University Graduate School of Medicine Tokyo Japan; ^5^ Faculty of Health Sciences Juntendo University Tokyo Japan; ^6^ Department of Rehabilitation Medicine Juntendo University Hospital Tokyo Japan

**Keywords:** cardiopulmonary exercise test, heart rate reserve, off‐pump coronary artery bypass grafting, peak oxygen uptake

## Abstract

**Background:**

Peak oxygen uptake (peak VO_2_) and heart rate reserve (HRR) are independent prognostic markers of cardiovascular disease. However, the impact of peak VO_2_ and HRR on long‐term prognosis after off‐pump coronary artery bypass grafting (OP‐CABG) remains unclear.

**Hypothesis:**

To determine the prognostic impact of peak VO_2_ and HRR in patients after OP‐CABG.

**Results:**

We enrolled 327 patients (mean age, 65.1 ± 9.3 years; male, 80%) who underwent OP‐CABG and participated in early phase II cardiac rehabilitation. All participants underwent cardiopulmonary exercise testing (CPET) at the beginning of such rehabilitation. Overall, 48 (14.6%) patients died during the median follow‐up period of 103 months. The non‐survivor had significantly lower levels of peak VO_2_ (10.6 ± 0.5 vs. 13.7 ± 0.2 ml/kg/min, p < .01) and HRR (24.2 ± 1.8 vs. 32.7 ± 0.8 beats/min, p < .01) than the survivor. In both groups, peak VO_2_ significantly correlated with HRR (p < .01). Moreover, patients were divided into four groups according to the peak VO_2_ and HRR levels for predicting total mortality. The low‐peak VO_2_/low‐HRR group had a significantly higher mortality risk than the other groups (hazards ratio, 5.61; 95% confidence interval, 2.59–12.16; p < .01). After adjusted the confounding factors, peak VO_2_ and HRR were independently associated with total mortality (both p < .05).

**Conclusions:**

HRR is a simple parameter of CPET and an important prognostic marker for the risk stratification of total mortality even in patients with low‐peak VO_2_ after OP‐CABG.

## 1 INTRODUCTION

Cardiopulmonary exercise testing (CPET) with peak oxygen uptake (peak VO_2_) measurement is increasingly performed in patients with cardiovascular disease. Peak VO_2_ is one of the superior measurements for assessing cardiovascular fitness and aerobic capacity.^1^ Consistently, peak VO_2_ is inversely and independently associated with cardiovascular events, cardiovascular mortality, and total mortality in large‐scale cohorts and patients with cardiovascular diseases.^2^, ^3^, ^4^

Currently, one of the established methods of coronary artery bypass grafting for patients with coronary artery disease is off‐pump coronary artery bypass grafting (OP‐CABG). OP‐CABG has been increasingly practiced in Japan, especially since 2000, with an annual increasing rate of >60%.^5^ However, cardiac autonomic function impairment and an increasing incidence rate of chronotropic incompetence have been observed in patients after OP‐CABG and on‐pump CABG as adverse effects of anesthesia methods and surgical techniques.^6^ OP‐CABG related factors such as thoracic content manipulation and post‐procedure bed rest contribute to the deleterious alterations in cardiac autonomic function.^6^

Heart rate response, which is a simple and easily obtained marker of cardiac autonomic function, is a powerful prognostic marker of coronary artery disease, independent of anti‐arrhythmic medication.^7^ Heart rate reserve (HRR) is a basic indicator of heart rate response that can predict the adverse outcomes of heart failure.^8^ However, the impact of peak VO_2_ and HRR on long‐term prognosis after OP‐CABG remains unclear. Therefore, we aimed to assess the prognostic impact of peak VO_2_ and HRR in patients after OP‐CABG.

## 2 METHODS

### 2.1 Study cohort

This was a retrospective study. We identified 327 consecutive patients who underwent OP‐CABG and participated in acute phase and early phase II cardiac rehabilitation at Juntendo University Hospital between July 2002 and February 2005. All eligible patients received standardized anesthesia and surgical management.^9^ The exclusion criteria consisted of uncontrolled arrhythmia that cause symptoms or hemodynamic compromise and uncontrolled symptomatic heart failure.^10^ In this study, diabetes mellitus was defined as a previous diagnosis, as shown in the medical records, with a hemoglobin A1c level of ≥6.5% or treatment with oral anti‐diabetic agents or insulin.^11^ Dyslipidemia was defined as a previous diagnosis with abnormal lipid profiles (triglyceride level ≥ 150 mg/dl, low‐density lipoprotein cholesterol level ≥ 140 mg/dl, or high‐density lipoprotein cholesterol level < 40 mg/dl) or treatment with anti‐dyslipidemic agents. In addition, hypertension was defined as a previous diagnosis with a systolic blood pressure of ≥140 mmHg and/or diastolic blood pressure of ≥90 mmHg or treatment with antihypertensive agents.^12^ All patients provided written informed consent, and the ethical committee of the institution approved this study.

### 2.2 Rehabilitation protocol

After undergoing OP‐CABG, all patients participated in acute phase and early phase II cardiac rehabilitation program. The early phase II cardiac rehabilitation exercise intensity was prescribed individually at the anaerobic threshold level, as measured by CPET using expiratory gas analysis or a rating of 11–13 on a standard Borg's perceived exertion scale, as we described previously.^13^

### 2.3 Measurements

Information on the clinical characteristics of the patients, including age, gender, risk factors, medical history, and concomitant use of medications, was obtained from their electronic medical records. We collected the data from the time closest to the start of the CR when multiple data were available for each patient. Laboratory data were collected after admission in the early morning after overnight fasting. At the start of cardiac rehabilitation from 6 to 8 days after OP‐CABG, we assessed the patients' anthropometric parameters, muscle strength, and exercise tolerance. Using the BOD POD (Life Measurement, Inc., Concord, CA), we measured bioelectrical impedance to determine the body fat and lean body mass. To measure peak VO_2_, patients underwent ergometric testing using Corival 400 (Lobe B.V. Groningen, Netherlands) with Vmax‐295 expiratory gas analyzer (SensorMedics Co., Yorba Linda, CA), as reported previously. After obtaining the resting heart rate in the sitting position during a 4 min rest, the patients warmed up for a few minutes at 20 W, followed by ramp loading (15 W/min) until they felt exhausted or experienced progressive angina, ST‐segment depression (≥2 mm), or sustained tachyarrhythmia. A standard 12‐lead electrocardiogram was continuously recorded. A satisfactory endpoint of CPET was characterized by a respiratory exchange ratio of greater than 1.10. The level of peak VO_2_ was defined as the highest peak VO_2_ achieved during the exercise, which was decided by well‐trained physicians who performed CPET on a clinical practice. We used the Cybex770 system (Cybex Division of Lumex, Ronkonkoma, NY) to measure the thigh muscle power.^14^, ^15^ Knee extensor and flexor muscles constitute the lower‐limb muscle strength, and the isokinetic peak torques of both muscles were measured at 60°/s and adjusted by body weight according to the following formula: strength (Nm) × 100/body weight (kg).^14^, ^15^ We also measured the grip strength, which represents the power of the handgrip. The HRR was calculated as the difference between peak and resting heart rates during CPET.

### 2.4 Follow‐up

After the initial assessment, all patients were followed up until the occurrence of total mortality or end of follow‐up (July 31, 2012). The outcome variable, total mortality, was ascertained from the electronic medical record, telephone call and patients letter. Total mortality was censored by cardiovascular and non‐cardiovascular deaths. All the study subjects were under continuous surveillance for the development of each of events.

### 2.5 Statistical analysis

All values are presented as mean ± SD. The groups were compared using the Student's *t*‐test, Mann–Whitney *U* test, or chi‐square test, as appropriate. Correlations between the CPET variables were assessed using Pearson's correlation coefficient. The association between variables and the end point of total mortality were assessed by univariate Cox proportional hazards analysis. Subsequently, parameters that significantly showed patients' prognosis according to univariate analysis were examined by multivariate Cox proportional hazards analysis using a stepwise forward method to assess their independent effects. To compare the analytical accuracy of the different parameters, we calculated the areas under the curve (AUC) for sensitivity and specificity through receiver‐operating characteristic (ROC) analysis. The four‐group combined peak VO_2_ and HRR were analyzed using one‐way ANOVA for continuous variables and the chi‐square test for categorical variables. Data were analyzed using Statistical Package for Social Sciences software, version 23.00 (Statistical Package for Social Sciences Inc, Chicago, IL). Statistical significance was defined as p < .05.

## 3 RESULTS

### 3.1 Clinical characteristics between the survivor and non‐survivor groups

**FIGURE 1:**
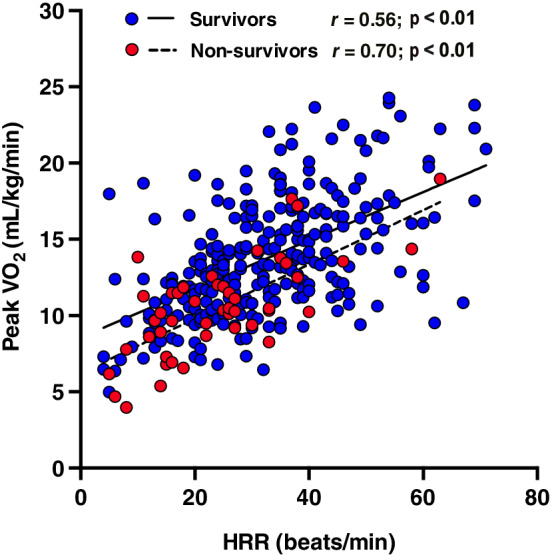
Correlation between peak VO_2_ and HRR. HRR, heart rate reserve; Peak VO_2_, peak oxygen uptake

The mean age of all patients was 65.1 ± 9.3 years, and 264 (80%) of them were males. The median and maximum follow‐up duration were 103 and 122 months, respectively. The survivor group was composed of 279 patients (85%), whereas the non‐survivor group was composed of 48 patients (15%). Eighteen patients (38%) had a cardiovascular death, and 30 (62%) had a non‐cardiovascular death. Table 1 summarizes the patients' characteristics of the study populations. The non‐survivor group was significantly older, had significantly lower levels of body mass index, hemoglobin, and albumin, and had significantly higher creatinine levels than the survivor group. The non‐survivor group also had lower levels of body fat mass, lean body mass, lower‐limb muscle strength, and grip strength than the survivor group. In addition, the levels of peak VO_2_ and HRR were significantly lower in the non‐survivor group than in the survivor group. After correlation analysis, the peak VO_2_ levels significantly correlated with HRR in the survivor (*r* = 0.56, p < .01) and non‐survivor (*r* = 0.70, p < .01) groups (Figure 1).

**TABLE 1:** Clinical characteristics

Characteristics	Survivors (n = 279)	Non‐survivors (n = 48)	p value
Age (years)	63.5 ± 9.2	68.9 ± 9.0	<.01
Male (%)	228 (81)	36 (75)	.28
BMI (kg/m^2^)	23.6 ± 2.6	21.9 ± 3.1	<.01
Hypertension (%)	190 (70)	34 (72)	.73
Dyslipidemia (%)	182 (68)	28 (58)	.21
Diabetes mellitus (%)	143 (53)	29 (60)	.33
Smoking (%)	120 (45)	22 (51)	.48
Family history of CVD (%)	60 (24)	12 (27)	.58
Prior myocardial infraction (%)	59 (23)	14 (35)	.10
Laboratory data			
Hemoglobin (g/dl)	13.3 ± 1.4	11.8 ± 1.7	<.01
Albumin (g/dl)	4.2 ± 0.4	3.9 ± 0.5	<.01
Creatinine (mg/dl)	1.1 ± 1.4	1.9 ± 2.1	<.01
Triglyceride (mg/dl)	143 ± 76	130 ± 76	.84
LDL‐C (mg/dl)	112 ± 37	116 ± 40	.70
HDL‐C (mg/dl)	47 ± 12	50 ± 16	.53
Fasting plasma glucoses (mg/dl)	137 ± 56	135 ± 51	.48
HbA1c (%)	6.2 ± 1.4	6.0 ± 1.2	.35
Diseased vessel			
1–2 vessel disease (%)	71 (29)	11 (26)	.89
3 vessel disease (%)	126 (51)	24 (57)	.41
Left main trunk (%)	50 (20)	5 (17)	.22
LVEF (%)	60 ± 16	56 ± 15	.93
Medications			
Aspirin (%)	264 (95)	45 (96)	.90
ACE‐I/ARB (%)	32 (11)	3 (6)	.29
β‐blockers (%)	67 (24)	12 (26)	.84
Statin (%)	57 (17)	9 (15)	.76
Oral anti‐diabetic (%)	73 (26)	11 (23)	.46
Insulin (%)	44 (16)	8 (17)	.84
Anthropometric parameters			
Body fat mass (kg)	12.7 ± 4.6	10.2 ± 5.0	<.01
Lean body mass (kg)	49.4 ± 8.5	44.8 ± 6.9	<.01
Muscle strength			
Lower limb muscle strength (Nm/kg × 100)	143.1 ± 41.7	112.1 ± 43.3	<.01
Grip strength (kg)	30.0 ± 7.9	22.0 ± 9.3	<.01
Exercise capacity			
Peak VO_2_ (ml/kg/min)	13.7 ± 3.9	10.6 ± 3.1	<.01
HRR (beats/min)	32.7 ± 13.9	24.2 ± 12.4	<.01

Abbreviations: ACE‐I, angiotensin‐converting enzyme inhibitors; ARB, angiotensin receptor blocker; BMI, body mass index; CVD, cardiovascular disease; HbA1c, hemoglobin A1c; HDL‐C, high‐density lipoprotein cholesterol; HRR, heart rate reserve; LDL‐C, low‐density lipoprotein cholesterol; LVEF, left ventricular ejection fraction; Peak VO_2_, peak oxygen consumption.

### 3.2 Cumulative event‐free survival rates based on peak VO_2_ levels and HRR

**FIGURE 2:**
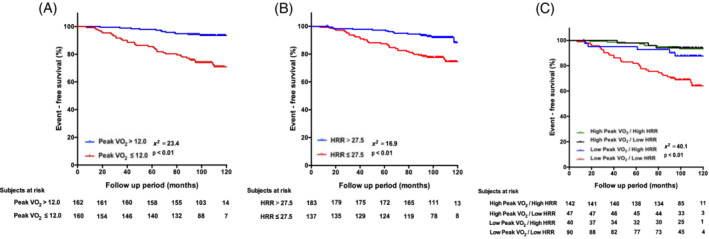
Prognostic implication of CPET variables. Event free survival shown by Kaplan–Meier method. (A) Peak VO_2_. (B) HRR. (C) Combined model of peak VO_2_ and HRR. CPET, cardiopulmonary exercise testing; HRR, heart rate reserve; Peak VO_2_, peak oxygen uptake

We performed ROC analysis to determine the optimal cutoff values on peak VO_2_ and HRR for indicating total mortality. ROC curve analysis estimated that peak VO_2_ cutoff value of 12.0 ml/kg/min yielded a sensitivity of 75% and a specificity of 65% (AUC, 0.74; 95% confidence interval, 0.67–0.81; p < .01), while the HRR cutoff value of 27.5 beats/min yielded a sensitivity of 71% and specificity of 67% (AUC, 0.69; 95% confidence interval, 0.61–0.77; p < .01). According to Kaplan–Meier curves, patients with low levels of peak VO_2_ (hazard ratio 4.84; 95% confidence interval, 2.34–9.99; p < .01) and HRR (hazard ratio, 3.34; 95% confidence interval, 1.79–6.24; p < .01) had higher risks of total mortality than those with high levels (Figure 2(A),(B)).

Moreover, we divided the patients into four groups according to each cutoff value as follows: Low peak VO_2_/low HRR, low peak VO_2_/high HRR, high peak VO_2_/low HRR, and high peak VO_2_/high HRR groups. Table 2 shows the clinical characteristics of the four groups. The age, male ratio, body mass index, hypertension prevalence, hemoglobin level, creatinine level, lean body mass, lower limb muscle strength, grip strength, peak VO_2_, peak heart rate, and HRR were significantly different among the four groups (all p < .05). The low peak VO_2_/low HRR group showed a high risk of total mortality (Figure 2(C)). The multivariate Cox regression analysis revealed that the low peak VO_2_/low HRR group had a significantly higher risk of total mortality than the high peak VO_2_/high HRR group after adjustment for age, gender, body mass index, grip strength, and hemoglobin, which were significant variables in the univariate analysis (hazard ratio, 3.62; 95% confidence interval, 1.08–12.12; p = .03; Supplemental Table 1). Furthermore, univariate and multivariate Cox hazard analyses were performed using peak VO_2_ and HRR. According to univariate Cox hazard analysis, age (hazard ratio, 1.05; 95% confidence interval, 1.02–1.09; p < .01), body mass index (hazard ratio, 0.80; 95% confidence interval, 0.72–0.90; p < .01), lean body mass (hazard ratio, 0.94; 95% confidence interval, 0.91–0.97; p < .01), hemoglobin (hazard ratio, 0.62; 95% confidence interval, 0.51–0.76; p < .01), creatinine (hazard ratio, 1.17; 95% confidence interval, 1.06–1.29; p < .01), low‐density lipoprotein cholesterol (hazard ratio, 1.01; 95% confidence interval, 1.00–1.03; p < .01), peak VO_2_ (hazard ratio, 0.79; 95% confidence interval, 0.93–0.98; p < .001), and HRR (hazard ratio, 0.95; 95% confidence interval, 0.93–0.98; p < .01) correlated significantly with total mortality (Table 3). Considering the high correlation between HRR and peak VO_2_ (Figure 1), the multivariate Cox hazard analysis was performed separately. After the confounding factors were adjusted, peak VO_2_ (hazard ratio, 0.75; 95% confidence interval, 0.64–0.84; p < .01) and HRR (hazard ratio, 0.96; 95% confidence interval, 0.92–0.99; p < .05) were independently associated with total mortality (Table 3(A),(B)).

**TABLE 2:** Clinical parameters among the four groups

Characteristics	Low peak VO_2_/low HRR	Low peak VO_2_/high HRR	High peak VO_2_/low HRR	High peak VO_2_/high HRR	p value
	(n = 91)	(n = 41)	(n = 49)	(n = 142)	
Age (years)	67.9 ± 8.7	66.5 ± 8.8	67.2 ± 9.1	62.1 ± 9.1	<.01
Male (%)	65 (71.4)	24 (58.5)	43 (87.8)	130 (91.5)	<.01
BMI (kg/m^2^)	22.7 ± 2.8	24.3 ± 2.9	23.0 ± 2.6	23.5 ± 2.5	<.01
Hypertension (%)	71 (82)	31 (76)	29 (59)	91 (66)	.02
Dyslipidemia (%)	98 (73)	29 (71)	28 (57)	52 (59)	.09
Diabetes mellitus (%)	49 (55)	25 (61)	29 (59)	67 (49)	.46
Smoking (%)	47 (56)	15 (38)	31 (66)	68 (51)	.05
Family history of CVD (%)	38 (29)	9 (22)	8 (17)	16 (20)	.24
Prior myocardial infraction (%)	24 (34)	9 (28)	5 (13)	24 (22)	.08
Laboratory data					
Hemoglobin (g/dl)	12.5 ± 1.7	12.9 ± 1.5	13.4 ± 1.4	13.5 ± 1.4	<.01
Albumin (g/dl)	4.0 ± 0.4	4.1 ± 0.3	4.1 ± 0.6	4.2 ± 0.4	.29
Creatinine (mg/dl)	1.9 ± 2.7	0.9 ± 0.3	1.2 ± 1.3	0.9 ± 0.4	<.01
Triglyceride (mg/dl)	153 ± 88	147 ± 70	131 ± 78	136 ± 69	.38
HDL‐C (mg/dl)	47 ± 14	49 ± 13	46 ± 13	48 ± 12	.92
LDL‐C (mg/dl)	110 ± 37	113 ± 38	106 ± 36	115 ± 38	.32
Fasting plasma glucoses (mg/dl)	138 ± 56	135 ± 48	138 ± 55	135 ± 56	.99
HbA1c (%)	6.2 ± 1.4	6.4 ± 1.4	6.2 ± 1.3	6.1 ± 1.5	.84
Diseased vessels					
1–2 Vessel disease (%)	21 (26)	9 (23)	10 (24)	42 (34)	.50
3 Vessel Disease (%)	47 (59)	20 (51)	24 (57)	59 (46)	.50
Left main trunk (%)	12 (15)	10 (25)	7 (19)	25 (20)	.56
LVEF (%)	56 ± 15	58 ± 17	61 ± 11	62 ± 17	.16
Medications					
Aspirin (%)	85 (96)	38 (93)	46 (94)	136 (97)	.73
ACE‐I/ARB (%)	5 (6)	7 (17)	4 (8)	11 (8)	.17
β‐blockers (%)	25 (28)	11 (27)	8 (16)	33 (23)	.82
Statin (%)	20 (23)	8 (20)	10 (20)	17 (12)	.18
Oral anti‐diabetic agents (%)	13 (15)	9 (22)	13 (27)	25 (18)	.35
Insulin (%)	15 (17)	9 (22)	8 (16)	20 (14)	.70
Anthropometric parameters					
Body fat mass (kg)	11.4 ± 5.1	13.9 ± 4.9	12.5 ± 4.8	12.3 ± 4.3	.05
Lean body mass (kg)	46.6 ± 9.1	46.3 ± 7.7	48.8 ± 8.5	51.0 ± 7.6	<.01
Muscle strength					
Lower limb muscle strength (Nm/kg × 100)	119.6 ± 36.8	121.2 ± 39.3	143.6 ± 40.0	160.5 ± 42.2	<.01
Grip strength (kg)	23.9 ± 9.2	25.5 ± 9.0	29.1 ± 7.4	32.5 ± 6.8	<.01
CPET parameters					
Post operation days	7.1 ± 3.2	6.4 ± 2.1	6.5 ± 5.5	6.3 ± 3.4	.77
Peak VO_2_ (mL/kg/min)	9.5 ± 1.9	10.2 ± 1.2	13.9 ± 1.9	16.3 ± 3.1	<.01
Resting HR (beats/min)	88.9 ± 14.1	83.4 ± 11.6	90.1 ± 12.5	88.3 ± 11.8	.06
Peak HR (beats/min)	107.1 ± 15.8	120.4 ± 14.4	111.3 ± 13.4	129.7 ± 12.6	<.01
HRR (beats/min)	18.0 ± 6.2	38.9 ± 13.0	21.0 ± 5.4	41.4 ± 9.9	<.01
Work rate (W)	50.6 ± 16.9	59.9 ± 15.1	70.5 ± 19.5	87.4 ± 23.6	<.01
ΔVO_2_/ΔWork rate (ml/min/W)	5.72 ± 2.8	5.87 ± 2.3	8.42 ± 5.3	8.79 ± 3.2	<.01

Abbreviations: ACE‐I, angiotensin‐converting enzyme inhibitors; ARB, angiotensin receptor blocker; BMI, body mass index; CPET, cardiopulmonary exercise test; CVD, cardiovascular disease; HbA1c, hemoglobin A1c; HDL‐C, high‐density lipoprotein cholesterol; HRR, heart rate reserve; HR, heart rate; LDL‐C, low‐density lipoprotein cholesterol; LVEF, left ventricular ejection fraction; Peak VO_2_, peak oxygen uptake.

**TABLE 3:** Univariate and multivariate analysis on total mortality

Variables	Univariate		Multivariate	
	HR (95%)	p value	HR (95%)	p value
A: Adjusted factors including peak VO_2_				
Age (years)	1.05 (1.02‐1.09)	<.01		
BMI (kg/m^2^)	0.80 (0.72‐0.90)	<.01		
Lean body mass (kg)	0.94 (0.91‐0.97)	<.01		
Hemoglobin (g/dl)	0.62 (0.51‐0.76)	<.01		
Creatinine (mg/dl)	1.17 (1.06‐1.29)	<.01		
LDL‐C (mg/dl)	1.01 (1.00‐1.03)	0.01		
Peak VO_2_ (ml/kg/min)	0.79 (0.72‐0.86)	<.01	0.75 (0.64‐0.84)	<.01
B: Adjusted factors including HRR				
Age (years)	1.05 (1.02‐1.09)	<.01		
BMI (kg/m^2^)	0.80 (0.72‐0.90)	<.01		
Lean body mass (kg)	0.94 (0.91‐0.97)	<.01		
Hemoglobin (g/dl)	0.62 (0.51‐0.76)	<.01		
Creatinine (mg/dl)	1.17 (1.06‐1.29)	<.01		
LDL‐C (mg/dl)	1.01 (1.00‐1.03)	<.01		
HRR (beats/min)	0.95 (0.93‐0.98)	<.01	0.96 (0.92‐0.99)	.03

Abbreviations: BMI, body mass index; HRR, heart rate reserve; LDL‐C, low‐density lipoprotein cholesterol; Peak VO_2_, peak oxygen uptake.

## 4 DISCUSSION

This study demonstrated that peak VO_2_ and HRR were significantly associated with total mortality risk in patients who underwent OP‐CABG. In addition, combined peak VO_2_ and HRR was an important prognostic marker, especially in patients with low‐peak VO_2_. To our knowledge, this report is the first to demonstrate the clinical usefulness of HRR to identify high‐risk patients after OP‐CABG.

Peak VO_2_ is widely used for assessing exercise capacity and provides prognostic information for patients with cardiovascular diseases.^16^, ^17^ Stewart et al. reported that poor exercise capacity has increased early and 5‐year mortality with on pump‐CABG, whereas those with better exercise capacity have improved survival with on pump‐CABG.^18^ Similarly, the present study revealed that a low‐peak VO_2_ level was associated with mortality in patients who underwent OP‐CABG (Figure 2(A)). Exercise capacity is generated through a complex interaction among the cardiovascular, respiratory, and muscular systems. However, exercise capacity impairment as well as increased total mortality may occur because of physiologic conditions such as aging, and pathologic conditions including muscular weakness and fatigue.^17^, ^19^, ^20^

When a healthy human performs maximal aerobic exercise, VO_2_ increases by approximately 7.7‐fold. This increment is achieved by 2.5‐fold increase in heart rate, a 2.5‐fold increase in arteriovenous oxygen difference, and a 1.4‐fold increase in stroke volume.^21^ Thus, heart rate increase is one of the strongest contributor to one's ability to sustain aerobic exercise.^22^ Patients with a low increase in heart rate on treadmill‐exercise echocardiography and apparently healthy subjects with a low increase in heart rate during exercise have poor long‐term outcomes.^7^, ^23^, ^24^ Niemela et al. evaluated a similar significance in patients who underwent on pump‐CABG and demonstrated that on pump‐CABG causes a marked attenuation of heart rate variability, but the prognostic significance of this attenuation is unknown.^25^

From this point, this finding agrees with our results; that is, impaired HRR was associated with increased total mortality in patients who underwent OP‐CABG. As an important indicator of chronotropic incompetence, HRR was associated with mortality in the patients with coronary artery disease and the healthy subjects.^7^, ^24^ The mechanism underlying the importance of HRR on the risk of mortality maybe related to not only chronotropic incompetence, but also a complex interplay among several factors such as age, gender, physical conditioning, and venous return.^26^

The present study showed that both low levels of peakVO_2_ and HRR were independent factor of poor prognosis in separate model analysis. After a simple combination of peak VO_2_ and HRR, patients with high‐peak VO_2_ maintained a good survival rate despite having an impaired HRR.^27^, ^28^, ^29^ However, the low‐peak VO_2_/low‐HRR group clearly showed the lowest survival rate compared with the low‐peak VO_2_/high‐HRR group (Figure 2(C)). As the age advances, the mitochondrial activity declines, which lead to a decrease in cellular oxygen delivery. In addition, as described previously, gender and physical conditioning also effects the deterioration of HRR. These complex factors might be associated with the poor prognosis of the low‐peak VO_2_ /low‐HRR group. The main clinical implication of this study is that HRR, a simple parameter measured in CPET, may be useful for identifying high‐risk subjects especially among patients with low‐peak VO_2_ after OP‐CABG. These findings may be important in clinical practice.

### 4.1 Study limitations

This study has several limitations. First, it was observational character with relatively small sample size, single center study, only Japanese subjects, and low‐event rate. Second, the data of physical activity and other health behaviors after the exercise test were lacking. Third, this analysis was limited to patients who participated in the cardiac rehabilitation program. Fourth, the dosage of β‐blockers and the heart rate variability were not included in our data. Finally, the exercise tests were performed around 1 week after CABG. The estimates in the present analysis might not be relevant if the exercise test was performed weeks to months post CABG, which is more likely the case in other institutions. Therefore, further studies are needed to confirm our study results and determine whether treatment intensity modification influences the HRR and prognosis of patients.

## 5 CONCLUSION

In conclusion, HRR, which is a simple parameter of CPET, was an important prognostic marker for the risk stratification of total mortality even in patients with low‐peak VO_2_ after OP‐CABG.

## CONFLICT OF INTEREST

The authors declare that there is no conflict of interest.

## Supporting information


**Supplemental Table 1** Multivariate analysis on total mortality among the four groups
