# Supplementary material for: Prognostic impact of peak oxygen uptake and heart rate reserve in patients after off‐pump coronary artery bypass grafting
Source: Clin Cardiol. 2021 Feb 25;44(4):580–7. doi: 10.1002/clc.23579 (PMC8027571; doi:10.1002/clc.23579)
Supplement: Supplementary file 1 — Supplemental Table 1 Multivariate analysis on total mortality among the four groups [file CLC-44-580-s001.docx]

| **Variables** | **Multivariate** | |
| --- | --- | --- |
|  | **HR (95%)** | **p Value** |
| High peak VO_2_ / High HRR | Reference |  |
| High peak VO_2_ / Low HRR | 1.9 (0.40-8.56) | 0.53 |
| Low peak VO_2_ / High HRR | 0.39 (0.04-3.57) | 0.41 |
| Low peak VO_2_ / Low HRR | 3.62 (1.08-12.12) | 0.03 |

**Supplemental TABLE 1**

**Multivariate analysis on total mortality among the four groups**

Abbreviations: HRR, heart rate reserve; Peak VO_2_, peak oxygen uptake.

Adjusted variables: age, gender, body mass index, grip strength, and hemoglobin.
